# Recent immigrants alter the quantitative genetic architecture of paternity in song sparrows

**DOI:** 10.1002/evl3.162

**Published:** 2020-02-25

**Authors:** Jane M. Reid, Peter Arcese

**Affiliations:** ^1^ Centre for Biodiversity Dynamics NTNU Trondheim Norway; ^2^ School of Biological Sciences University of Aberdeen Aberdeen United Kingdom; ^3^ Forest & Conservation Sciences University of British Columbia Vancouver British Columbia Canada

**Keywords:** Cross‐sex genetic correlation, dispersal, extra‐pair paternity, gene flow, genetic groups, immigration, indirect genetic effects, mating system, polyandry, quantitative genetics, sexual conflict, sexual selection

## Abstract

Quantifying additive genetic variances and cross‐sex covariances in reproductive traits, and identifying processes that shape and maintain such (co)variances, is central to understanding the evolutionary dynamics of reproductive systems. Gene flow resulting from among‐population dispersal could substantially alter additive genetic variances and covariances in key traits in recipient populations, thereby altering forms of sexual conflict, indirect selection, and evolutionary responses. However, the degree to which genes imported by immigrants do in fact affect quantitative genetic architectures of key reproductive traits and outcomes is rarely explicitly quantified. We applied structured quantitative genetic analyses to multiyear pedigree, pairing, and paternity data from free‐living song sparrows (*Melospiza melodia*) to quantify the differences in mean breeding values for major sex‐specific reproductive traits, specifically female extra‐pair reproduction and male paternity loss, between recent immigrants and the previously existing population. We thereby quantify effects of natural immigration on the means, variances, and cross‐sex covariance in total additive genetic values for extra‐pair paternity arising within the complex socially monogamous but genetically polygynandrous reproductive system. Recent immigrants had lower mean breeding values for male paternity loss, and somewhat lower values for female extra‐pair reproduction, than the local recipient population, and would therefore increase the emerging degree of reproductive fidelity of social pairings. Furthermore, immigration increased the variances in total additive genetic values for these traits, but decreased the magnitudes of the negative cross‐sex genetic covariation and correlation below those evident in the existing population. Immigration thereby increased the total additive genetic variance but could decrease the magnitude of indirect selection acting on sex‐specific contributions to paternity outcomes. These results demonstrate that dispersal and resulting immigration and gene flow can substantially affect quantitative genetic architectures of complex local reproductive systems, implying that comprehensive theoretical and empirical efforts to understand mating system dynamics will need to incorporate spatial population processes.

**Impact Summary**

Reproductive interactions among females and males, and resulting production of offspring, are critical processes that propagate genes across generations and thereby drive evolution. Evolutionary ecologists consequently aim to identify processes that shape reproductive outcomes, and particularly to understand what determines which males successfully sire females’ offspring. Paternity can be greatly affected by genes expressed in interacting females and males, and by associations between these genes across the sexes. Quantifying such cross‐sex genetic associations, and understanding how these associations are maintained and constrained, is therefore central to understanding the course of evolution.

Cross‐sex genetic associations can be shaped by processes acting within any focal population, including local natural selection and mate choice (and resulting “sexual selection”). But, they could also be substantially affected by genes imported by immigrants. Resulting “gene flow” could increase local genetic variation, and alter the critical cross‐sex genetic associations. However, such effects have not been explicitly quantified in wild animal populations.

Accordingly, we provide a framework for conceptualizing and estimating effects of immigrants on local genetic variation and cross‐sex genetic associations. We apply this framework to paternity data from free‐living song sparrows. These sparrows are socially monogamous but show substantial extra‐pair paternity; many offspring are sired by males other than a female's socially paired mate. We show that immigrants import genes that increase male paternity success and tend to decrease female infidelity; immigration therefore increased genetic pair fidelity in the recipient population. Furthermore, immigration weakened the cross‐sex genetic association between male paternity success and female infidelity, reducing the degree to which selection for male success would induce female infidelity. Our results illustrate how natural immigration can alter the genetic basis of complex reproductive systems, and imply that understanding evolutionary dynamics will require evolutionary ecologists to consider sexual selection in the context of large‐scale meta‐population systems.

The pattern of paternity that emerges across offspring of interacting females and males in any population constitutes the outcome of sexual selection, and determines which genes and genotypes pass to subsequent generations. Identifying processes that shape paternity outcomes is therefore central to understanding the evolutionary dynamics of reproductive traits and overall reproductive systems (Arnqvist and Kirkpatrick 2005; Evans and Simmons 2008; Parker and Birkhead 2013).

In general, paternity depends on multiple precopulatory and postcopulatory traits expressed by females and males (Evans and Simmons 2008; Parker and Birkhead 2013). These traits are themselves shaped by additive genetic variances and covariances evident within and between the sexes, and emerging forms of indirect selection and sexual conflict. Major steps toward understanding paternity outcomes and associated trait and reproductive system evolution are therefore to quantify key additive genetic variances and cross‐sex covariances and, furthermore, to identify how such variances and covariances are modulated and maintained (Mead and Arnold 2004; Kruuk et al. 2008; Evans 2010; Long et al. 2012; Gosden and Chenoweth 2014; Connallon and Hall 2016; Travers et al. 2016; Cox et al. 2017; Reid and Wolak 2018; Connallon and Matthews 2019; Stepanacz and Houle 2019).

Cross‐sex genetic covariances can reflect pleiotropy and/or physical linkage among genes that affect female and male traits, and can also result from patterns of nonrandom mating and paternity allocation that emerge from the reproductive system and generate statistical linkage disequilibria (e.g., Mead and Arnold 2004; Bonduriansky and Chenoweth 2009; Gosden and Chenoweth 2014; Bocedi and Reid 2015). However, local genetic and phenotypic means, variances, and covariances in reproductive traits could also be substantially shaped by genes imported by immigrants to a focal population (e.g., Guillaume and Whitlock 2007). Indeed, in general, gene flow can be one major driver of, or constraint on, adaptive evolution (Lenormand 2002; Garant et al. 2007). In the context of reproductive systems, if there is local adaptation in reproductive and sexually selected traits (e.g., Candolin 2019), immigration and resulting gene flow could cause substantial divergence from local naturally and sexually selected trait optima (Day 2000; Lenormand 2002; Garant et al. 2007; Long et al. 2012; Connallon et al. 2018). Immigration could therefore increase local genetic variation, and alter cross‐sex genetic covariances from those arising solely given the local reproductive system. Specifically, by pulling genetic values in both sexes away from their current local means, introgressive gene flow could transform existing cross‐sex genetic covariances in the original native and/or immigrant populations into smaller, larger, or opposite‐sign covariances in the contemporary admixed population (e.g., Long et al. 2012; Connallon and Hall 2016). Immigration could thereby shape reproductive systems and resulting paternity outcomes by directly altering trait means, and also induce further major changes by altering forms of indirect selection and sexual conflict that are shaped by cross‐sex genetic covariances. However, despite these expectations, the degree to which such gene flow can in practice alter the quantitative genetic architecture of key traits that shape reproductive systems and resulting paternity outcomes has rarely been quantified, especially in free‐living populations experiencing natural immigration (Guillaume and Whitlock 2007; Long et al. 2012).

One outcome that emerges from reproductive interactions among females and males is the degree of extra‐pair (or extra‐group) paternity occurring in socially monogamous or group‐living species. Here, a focal female's offspring could be sired by her socially paired male(s), or by other male(s) in the population (Fig. 1). Such extra‐pair or extra‐group paternity is taxonomically widespread (e.g., Jennions and Petrie 2000; Neff and Gross 2001; Griffith et al. 2002; Laloi et al. 2009; Nichols et al. 2015) and could affect additive and nonadditive genetic values of offspring (Reid and Sardell 2011; Reid et al. 2015), distributions of male versus female reproductive success and population relatedness structure (Webster et al. 1995; Lebigre et al. 2012; Germain et al. 2018), and the degree of paternal care (Neff and Gross 2001; Arnqvist and Kirkpatrick 2005; Gow et al. 2019). The evolving occurrence of extra‐pair paternity could thereby profoundly affect overall social systems and evolutionary outcomes (Kokko 1999). Yet, the quantitative genetic drivers and constraints on extra‐pair paternity are still far from clear (Reid et al. 2011, 2014a, 2014c; Reid 2012, 2014; Parker and Birkhead 2013).

**Figure 1:**
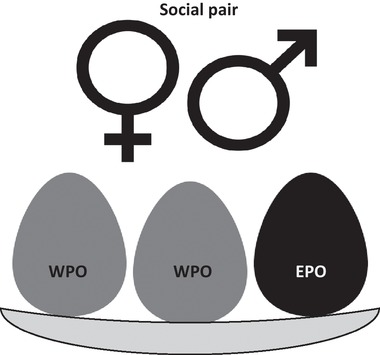
Illustration of the occurrence of extra‐pair paternity within a breeding attempt, viewed as a single joint “emergent” trait expressed by a focal socially paired female and male. In this example, two offspring are sired by the female's socially paired male (gray, within‐pair offspring, WPO), and one offspring is sired by an extra‐pair male (black, extra‐pair offspring, EPO). The degree of extra‐pair paternity observed at the brood level (in this case 1/3) is the focal binomial trait and can be envisaged as a joint outcome of two sex‐specific latent traits: female liability for extra‐pair reproduction and male liability for paternity loss.

Extra‐pair paternity reflects expression of numerous underlying traits, including simultaneous polyandry (i.e., female mating with multiple males within a reproductive episode) and subsequent postcopulatory female choice, alongside mating frequency and postcopulatory siring success under sperm competition of a female's socially paired male (e.g., Jennions and Petrie 2000; Simmons 2005; Parker and Birkhead 2013). The paternity status of each offspring in a focal brood (i.e., extra‐pair or within‐pair) can therefore be viewed as a complex emergent trait, which is jointly shaped by numerous genetic and environmental effects of paired females and males, and is appropriately treated within the framework of evolutionary quantitative genetics (Arnqvist and Kirkpatrick 2005; Reid 2014; Reid et al. 2014a). Accordingly, diverse studies have attempted to explain the occurrence of extra‐pair paternity, and underlying polyandry, by quantifying components of indirect selection that could compensate for the widely postulated negative direct selection against female multiple mating (Jennions and Petrie 2000; Arnqvist and Kirkpatrick 2005; Simmons 2005). This includes testing the hypothesis that there is a positive cross‐sex genetic covariance between the degree of female polyandry or extra‐pair reproduction and male siring success and hence reproductive success (Evans and Simmons 2008; Forstmeier et al. 2011, 2014; Egan et al. 2016; Travers et al. 2016; Reid and Wolak 2018). However, empirical evidence of such covariances is still scant, and their emergence solely due to nonrandom mating might commonly be impeded by other properties of mating and reproductive systems (Reid et al. 2014a; Evans and Simmons 2008; Bocedi and Reid 2015; Reid and Wolak 2018). Furthermore, attempts to explain observed reproductive systems and paternity outcomes solely based on local selection will be incomplete, and potentially misleading, if patterns of dispersal mean that immigrants import genes that alter additive genetic means, variances, and cross‐sex covariances in and among key reproductive traits that affect paternity outcomes.

When reproductive traits expressed by females and males are at least partly autosomal, immigrants of both sexes can potentially import genes that affect means, variances, and covariances in and among traits expressed by each sex. Specifically, both female and male immigrants could import genes that affect polyandry, male mating frequency, postcopulatory processes, and consequent paternity outcomes. Quantifying full genetic effects of immigration on the overall reproductive system therefore requires quantifying genetic effects of immigrants of both sexes on paternity outcomes across descendants of both sexes.

Accordingly, we first present a general conceptual and analytical quantitative genetic framework for quantifying immigrant effects on means, variances, and cross‐sex covariances in additive genetic values. We highlight how this approach can be applied to extra‐pair paternity, which constitutes one key emergent trait that characterizes reproductive systems. We then implement such analyses using multigeneration pedigree, pairing, and paternity data from song sparrows (*Melospiza melodia*). We thereby quantify effects of gene flow resulting from immigration on additive genetic means, variances, and covariances in female extra‐pair reproduction and male paternity loss relative to the existing local population. In doing so, we provide a general framework for understanding how natural immigration can alter the quantitative genetic architecture of the local reproductive system or other emergent traits, and illustrate how quantitative genetic consequences of immigration can now be explicitly evaluated in wild populations.

## Conceptual Framework

### QUANTITATIVE GENETIC ANALYSIS OF IMMIGRANT EFFECTS

“Animal models” (i.e., generalized linear mixed models that utilize information on relatedness among phenotyped individuals to partition genetic and environmental variances in focal traits) facilitate estimation of quantitative genetic parameters in wild populations in the absence of structured breeding designs (Kruuk 2004; Charmantier et al. 2014). Extension to “genetic groups animal models” allows explicit estimation of additive genetic effects of immigrants (reviewed by Wolak and Reid 2017; demonstrated in a wild population by Wolak et al. 2018). Briefly, standard pedigree‐based animal models estimate additive genetic (co)variances in a defined base population comprising “phantom parents” of observed individuals with unknown parents. Given complete pedigree data for a focal population and study period with no immigration, these individuals comprise the parents of the initially observed population (hereafter “founders”). With immigration, the base population can be split into two (or more) genetic groups, comprising phantom parents of recent immigrants versus pre‐existing founders. The difference in mean breeding value for any focal trait between these groups, *g*, can then be estimated (Quaas 1988; Westell et al. 1988; Wolak and Reid 2017).

Such genetic groups animal models state that an individual's total additive genetic value *u*_i_ for any trait equals its breeding value *a*_i_ for that trait (i.e., the deviation from the defined group mean due to additive effects of the individual's genes) plus the product of the mean immigrant genetic group effect *g* and the proportion of the focal individual's genome *q*_i_ that is expected to have originated from the immigrant genetic group (Quaas 1988; Westell et al. 1988; Wolak and Reid 2017). Specifically:

(1) $$u_{i}=a_{i}+g\cdot q_{i}.$$

The value of *u*_i_ reflects that both breeding values (*a*_i_) and genetic group contributions (*q*_i_) are inherited by offspring from parents following standard assumptions of the infinitesimal model of quantitative genetics (i.e., invoking numerous underlying loci, Barton et al. 2017). Consequently, *u*_i_ measures the deviation from the overall population mean due to additive effects of an individual's genes. The value of *g*, which is the fixed difference between defined genetic group means, can be directly estimated as the slope of a linear regression of phenotype on *q*_i_ fitted across individuals within the animal model. Individual values of *q*_i_ can be directly calculated from pedigree data (Quaas 1988; Westell et al. 1988; Wolak and Reid 2017). Such genetic groups animal models directly estimate variances and covariances in *a*_i_ (i.e., additive genetic (co)variances) in and among focal traits, assuming these (co)variances are equal for the phantom parents of the founder and immigrant genetic groups (Wolak and Reid 2017, although see Muff et al. 2019). Here, we show that variances and covariances in *u*_i_ can then be calculated for observed or hypothetical populations comprising admixed descendants of defined founders and subsequent immigrants, thereby allowing evaluation of effects of immigration on quantitative genetic architectures of focal traits.

Specifically, given any two traits expressed by females and males (subscripts f and m, respectively), appropriately specified genetic groups animal models can directly estimate the additive genetic variances Var(*a*_f_) and Var(*a*_m_), the cross‐sex additive genetic covariance cov(*a*_f_,*a*_m_), and the mean genetic group effect on each trait (i.e., fixed effects *g*_f_ and *g*_m_). The two traits could be sex‐limited phenotypes, as is often of interest in the context of reproductive systems, or could be the same phenotype expressed to different degrees (i.e., sexual dimorphism). The variances and covariance in total additive genetic values (Var(*u*_f_), Var(*u*_m_), and cov(*u*_f_,*u*_m_)) can then be calculated as functions of estimated (co)variances of the two underlying variables *a*_i_ and *q*_i_ and the fixed effect *g* (see eq. 1):

(2) $$\mathrm{Var}\left(u_{f}\right)=\mathrm{Var}\left(a_{f}\right)+{g_{f}}^{2}\cdot \mathrm{Var}\left(q\right),$$

with an analogous expression for Var(*u*_m_). Meanwhile,

(3) $$\mathrm{cov}\left(u_{f},u_{m}\right)=\mathrm{cov}\left(a_{f},a_{m}\right)+g_{f}\cdot g_{m}\cdot \mathrm{Var}\left(q\right).$$

Derivations and further explanations are in Supporting Information S1. These equations yield general insights into how immigration could potentially affect the variance and cross‐sex covariance in *u* relative to the variances and cross‐sex covariance in *a*, and hence the total genetic (co)variance. Equation (2) shows that immigration will increase Var(*u*) above Var(*a*) to a degree that depends on the magnitude of the difference in mean breeding value between the immigrant and founder groups (*g*, i.e., the degree of divergence between the local population and the immigrants’ source population(s)), and the variance in immigrant ancestry among individuals (Var(*q*)). As *g* ^2^, Var(*q*), and Var(*a*) cannot be negative, Var(*u*) cannot be less than Var(*a*). Equation (3) shows that immigration will alter cov(*u*_f_,*u*_m_) relative to cov(*a*_f_,*a*_m_) to a degree that depends on the signs and magnitudes of *g*_f_ and *g*_m_, the magnitude of Var(*q*), and the sign and magnitude of cov(*a*_f_,*a*_m_). Consequently, cov(*u*_f_,*u*_m_) could be larger or smaller than cov(*a*_f_,*a*_m_) with the same sign, or the sign could be reversed, potentially transforming a negative cross‐sex covariance in *a* into a positive cross‐sex covariance in *u* (or vice versa, Supporting Information S1).

### EXTRA‐PAIR PATERNITY AS AN EMERGENT QUANTITATIVE TRAIT

Extra‐pair paternity is a key emergent trait that allows female and male reproductive fitness to diverge, potentially weakening the positive cross‐sex genetic covariance for fitness that is otherwise likely to arise in socially monogamous reproductive systems. However, the full suite of behavioral, morphological, and physiological traits that affect paternity outcomes cannot feasibly be measured in any study. Understanding the maintenance and microevolution of extra‐pair paternity is therefore an ongoing challenge (Arnqvist and Kirkpatrick 2005; Reid et al. 2011, 2014a; Parker and Birkhead 2013; Reid 2014).

Consequently, one direct approach to evolutionary quantitative genetic dissection of the reproductive system is to define the paternity outcome for offspring produced by a focal social pair (i.e., within‐pair versus extra‐pair, Fig. 1) as the focal phenotype. This phenotype can be considered a joint “emergent” or “associative” trait that is primarily affected by genetic and environmental effects of two socially paired individuals: the female that could produce extra‐pair or within‐pair offspring, and the male that could lose or achieve paternity of each offspring he rears (Fig. 1, Reid et al. 2014a). Genetic (co)variance components for the single joint trait can then be estimated using variance partitioning methods, as employed for indirect genetic effects (e.g., Bijma et al. 2007; Bijma 2011, Supporting Information S2). Non‐Gaussian phenotypes can also be treated within the framework of quantitative genetics by considering genetic and environmental effects on implicit underlying latent traits that translate into observed outcomes and can be assumed to fulfill the fundamental assumption of multivariate normality (e.g., de Villemereuil et al. 2016).

Specifically, the occurrence of extra‐pair paternity reflects a female's liability for extra‐pair reproduction and her socially paired male's liability for paternity loss (Fig. 1; or, inversely, the occurrence of within‐pair paternity reflects a female's liability for within‐pair reproduction and the male's liability for paternity success, Reid et al. 2014a). Positive and negative cross‐sex genetic covariances would imply that females with high liability for extra‐pair reproduction have male relatives with high liability for paternity loss or paternity success, respectively. Note that such covariances refer to associations across opposite sex relatives, not solely across socially paired mates (Reid et al. 2014a).

The additive genetic variances in female and male liabilities (Var(*a*_f_) and Var(*a*_m_)) and the cross‐sex additive genetic covariance (cov(*a*_f_,*a*_m_)) can be estimated given sufficient observations of paternity outcomes across breeding attempts made by females and males of known and varying relatedness (see Methods section). The total additive genetic variance in liability for extra‐pair paternity (i.e., the joint emergent trait) that is available for selection can then be estimated as:

(4) $$\mathrm{Var}\left(a_{T}\right)=\mathrm{Var}\left(a_{f}\right)+2\mathrm{cov}\left(a_{f},a_{m}\right)+\mathrm{Var}\left(a_{m}\right),$$

following Bijma et al. (2007) and Bijma (2011). Further, effects of immigration on female and male liabilities, and hence on the evolving reproductive system, can be estimated by fitting a genetic groups animal model that distinguishes founders and recent immigrants and thereby estimates *g*_f_ and *g*_m_ alongside Var(*a*_f_), Var(*a*_m_), and cov(*a*_f_,*a*_m_). The quantities Var(*u*_f_), Var(*u*_m_), and cov(*u*_f_,*u*_m_) can then be calculated (eqs. 2 and 3), allowing calculation of the total variance in total additive genetic value as:

(5) $$\mathrm{Var}\left(u_{T}\right)=\mathrm{Var}\left(u_{f}\right)+2\mathrm{cov}\left(u_{f},u_{m}\right)+\mathrm{Var}\left(u_{m}\right).$$

## Methods

### STUDY SYSTEM

Long‐term pedigree, pairing, and paternity data from song sparrows inhabiting Mandarte island, British Columbia, Canada, allow the required analyses. Each spring, adults (age ≥1 year) form female–male social pairings that defend territories and care for dependent broods (Smith et al. 2006). Each pair typically rears up to three broods of one to four offspring per year. Each year since 1975, nests were monitored and all chicks surviving ca. 6 days posthatch were uniquely color ringed (Smith et al. 2006). Mandarte lies within a large song sparrow meta‐population and receives occasional immigrants (∼1 per year on average), which are caught and color ringed after arriving (Marr et al. 2002; Wolak et al. 2018). All adults are therefore identifiable by visual resighting, and the socially paired female and male attending each nest were documented (Smith et al. 2006; Reid et al. 2016). All chicks and adults ringed during 1993–2016 were blood‐sampled and genotyped at ∼160 microsatellite markers, allowing individuals’ genetic parents to be identified with very high individual‐level statistical confidence (Sardell et al. 2010; Nietlisbach et al. 2017). The paternity status of each sampled offspring in each brood (i.e., extra‐pair versus within‐pair), and hence each female's phenotypic extra‐pair reproduction per brood (i.e., number of extra‐pair versus total offspring) and her socially paired male's corresponding degree of paternity loss, can consequently be quantified (Fig. 1, Reid et al. 2014a; Sardell et al. 2010). The genetic parentage data were also used to compile complete, accurate, pedigree data for quantitative genetic analyses (Reid et al. 2014b; Nietlisbach et al. 2017; Wolak et al. 2018, Supporting Information S2).

These data demonstrate frequent extra‐pair paternity: during 1993–2008, ∼28% of sampled chicks had extra‐pair sires, affecting ∼44% of broods (Sardell et al. 2010). Previous quantitative genetic analyses estimated substantial additive genetic variance in female liability for extra‐pair reproduction (Var(*a*_f_)), and detectable additive genetic variance in male liability to lose (or achieve) paternity (Var(*a*_m_), Reid et al. 2011, 2014a). This raises the question of how such genetic variation is locally maintained, especially because male paternity success is expected to be under positive direct selection (Reid et al. 2014c). Further, the cross‐sex genetic covariance between female extra‐pair reproduction and male paternity success (cov(*a*_f_,*a*_m_)) was estimated to be weakly positive (i.e., negative genetic covariance between female extra‐pair reproduction and male paternity loss), but with substantial uncertainty that might partly reflect unknown structure in the data (Reid et al. 2014a). The hypothesis that local additive genetic means, variances, and covariances in female and male liabilities, and hence the emerging reproductive system of extra‐pair paternity, are shaped by genetic values of incoming immigrants, and hence by local gene flow, has not been tested in song sparrows or any other system.

### QUANTITATIVE GENETIC ANALYSES

We fitted a univariate animal model with the observed degree of extra‐pair paternity per brood as the focal binomial phenotype (Fig. 1), random additive genetic effects of the socially paired female and male that reared each brood with the direct cross‐product, and regressions on the female's and male's immigrant genetic group coefficients *q*_i_. We thereby estimated the additive genetic variances in female extra‐pair reproduction Var(*a*_f_) and male paternity loss Var(*a*_m_), the cross‐sex additive genetic covariance cov(*a*_f_,*a*_m_), and immigrant genetic group effects on each sex‐limited trait (*g*_f_ and *g*_m_, respectively; for further detail, see Supporting Information S2).

For current purposes, we defined the immigrant genetic group as the phantom parents of all immigrants that arrived and bred on Mandarte since 1989 and hence contributed directly or proximately to extra‐pair paternity phenotypes observed during 1993–2016 (i.e., the period of genetic paternity assignment) through their own reproduction or that of surviving offspring produced during 1989–1992. We defined the founder genetic group as the phantom parents of all other individuals with unknown parents remaining in the pedigree after pruning to informative phenotyped individuals and their known ancestors (Supporting Information S2). As for any wild population study, the observed “founders” presumably include genetic contributions from previous immigrants. Current analyses therefore estimate the additive genetic effects of recent immigrants relative to the effects of all previous population members with nonzero expected genetic contributions to the currently defined founders (see Discussion section).

We additionally fitted independent random individual female, male, social pair, and year effects to account for any nonindependence of extra‐pair paternity observed in multiple broods produced by individuals or pairs or within years that could potentially inflate estimates of additive genetic (co)variances, yielding estimates of permanent individual, pair, and year variances (Supporting Information S2). Because previous analyses showed that male paternity success increases with age (Reid et al. 2014a, c), we fitted a linear regression on male age. Further, as *q*_i_ inevitably increased across cohorts hatched during 1993–2016 (Supporting Information S2), we additionally fitted a linear regression on year (since 1993) to reduce the possibility that estimates of *g*_f_ and/or *g*_m_ could be inflated by any environmentally induced change in the degree of extra‐pair paternity across years (e.g., Postma and Charmantier 2007); however, results remained similar when this regression was removed. As our aim was to partition the total phenotypic variation, we fitted no further fixed effects (Supporting Information S2).

The genetic groups animal model was fitted in a Bayesian framework, facilitating estimation of latent‐scale quantitative genetic parameters for the focal binomial trait and propagation of uncertainty to derived parameters. Models were fitted using package MCMCglmm (Hadfield 2010) in R version 3.5.1 (R Core Team 2018), using logit link functions and relatively uninformative priors (Supporting Information S2). The required inverse relatedness matrix and vectors of *q*_i_ were computed from the pruned pedigree using standard algorithms (package nadiv, Wolak 2012; for full detail, see Wolak and Reid 2017; Supporting Information S2).

To evaluate immigrants’ effects on the variances and cross‐sex covariance in total additive genetic value for female extra‐pair reproduction and male paternity loss, we calculated the posterior distributions of Var(*u*_f_), Var(*u*_m_), and cov(*u*_f_,*u*_m_) using equations (2) and (3), taking Var(*q*) as a constant estimated across all phenotyped individuals (Supporting Information S1). We then calculated the total variances Var(*a*_T_) and Var(*u*_T_) (eqs. 4 and 5). To facilitate comparative analyses, posterior distributions of the cross‐sex genetic correlations cor(*a*_f_,*a*_m_) and cor(*u*_f_,*u*_m_) were calculated as the respective covariances divided by the geometric mean of the variances. Latent‐scale heritabilities were calculated as the focal additive genetic variance divided by the sum of all estimated variance components (de Villemereuil et al. 2016). Posterior distributions are summarized as posterior means and modes with 95% highest posterior density credible intervals (95% CI) are calculated across 2000 samples with autocorrelation <0.05. For regression slopes and cross‐sex genetic covariances and correlations, the percentage of posterior density that was negative was additionally extracted. Key quantities and abbreviations are presented in Tables 1 and 2.

**Table 1:** Posterior mean, mode, 95% credible interval (95% CI), and proportion of posterior density that was negative (%negative) for (A) estimated fixed effects and (co)variance components for female liability for extra‐pair reproduction and male liability for paternity loss estimated from a univariate animal model with immigrant and founder genetic groups, and (B) associated derived parameters. Estimated permanent individual female, male, pair, and year variances were small (Supporting Information S3)

(A) Estimated parameters	Mean [mode]	95% CI [%negative]
*Fixed effects*		
Intercept	–0.67 [–0.81]	–1.83 to 0.65
Immigrant genetic group effect on female liability (*g*_f_)	–1.17 [–0.99]	–2.90 to 0.46 [91.4%]
Immigrant genetic group effect on male liability (*g*_m_)	–1.55 [–1.53]	–3.07 to 0.04 [97.2%]
Year	0.05 [0.06]	–0.01 to 0.11 [3.7%]
Male age	–0.19 [–0.18]	–0.32 to –0.05 [99.9%]
*(Co)variance components*		
Additive genetic variance in female liability (Var(*a*_f_))	1.26 [1.12]	0.33 to 2.28
Additive genetic variance in male liability (Var(*a*_m_))	0.47 [0.38]	0.001 to 1.01
Additive genetic covariance (cov(*a*_f_,*a*_m_))	–0.37 –0.40]	–0.91 to 0.04 [94.7%]
Residual variance	3.58 [3.55]	2.54 to 4.55

		
(B) Derived parameters		
Additive genetic correlation (cor(*a*_f_,*a*_m_))	–0.51 [–0.69]	–0.98 to 0.01 [94.7%]
Heritability of female liability (*h_f_* ^2^)	0.20 [0.21]	0.07 to 0.35
Heritability of male liability (*h_m_* ^2^)	0.08 [0.05]	0.001 to 0.16
Total additive genetic variance (Var(*a*_T_))	0.98 [0.91]	0.09 to 2.03

**Table 2:** Posterior mean, mode, 95% credible interval (95% CI), and proportion of posterior density that was negative (%negative) for derived parameters for total additive genetic values for female liability for extra‐pair reproduction and male liability for paternity loss

	Mean [mode]	95% CI [%negative]
Variance in total genetic value for female liability (Var(*u*_f_))	1.39 [1.16]	0.34 to 2.41
Variance in total genetic value for male liability (Var(*u*_m_))	0.66 [0.50]	0.05 to 1.32
Covariance in total genetic value (cov(*u*_f_,*u*_m_))	–0.27 [–0.13]	–0.85 to 0.25 [83.6%]
Correlation in total genetic value (cor(*u*_f_,*u*_m_))	–0.28 [–0.28]	–0.81 to 0.28 [83.6%]
Total variance in total genetic value (Var(*u*_T_))	1.51 [1.11]	0.36 to 2.79

To illustrate the biological magnitude of estimated genetic effects of immigrants in altering the local mean extra‐pair paternity rate, and hence the reproductive system, we back‐transformed latent‐scale estimates of *g*_f_ and *g*_m_ to the observed phenotypic scale. We computed these effects for the middle year of the phenotypic dataset (2004) given mean male age across all observations (2.7 years), and compared the effect of mean observed *q* to *q* = 0 (representing an isolated population with no recent immigration). Finally, for purely illustrative purposes to aid conceptual insight, we computed and plotted posterior mean predicted values of *a*_fi_, *a*_mi_, *u*_fi_, and *u*_mi_ for all phenotyped individuals hatched between 2003 and 2012. Most of these individuals have phenotyped ancestors and descendants and numerous contemporary relatives, meaning that predicted breeding values are likely to be relatively little biased toward an individual's own phenotype.

## Results

**Figure 2:**
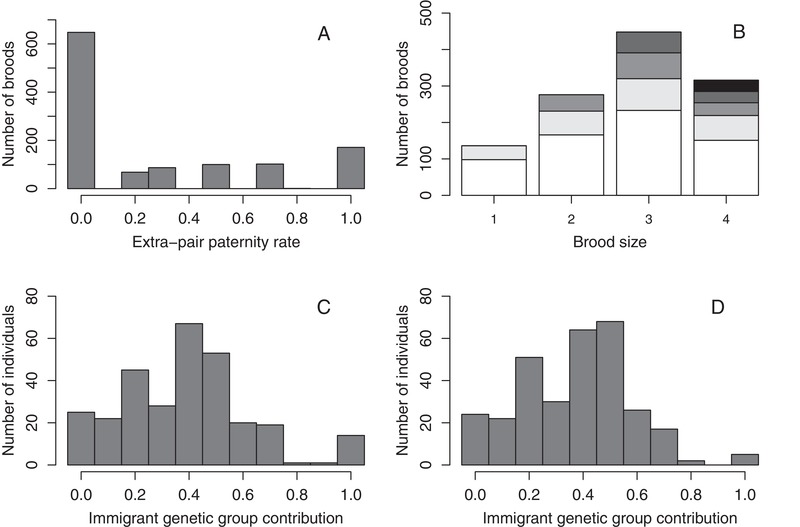
Distributions of observed extra‐pair paternity rate shown as (A) a proportion (mean 0.288 ± 0.371 SD) and (B) a binomial trait as analyzed across 1177 observed song sparrow broods, and the expected immigrant genetic group contributions (*q*_i_) to (C) 295 phenotyped females (mean 0.39 ± 0.24 SD) and (D) 309 phenotyped males (mean 0.38 ± 0.20 SD). Mean contributions across 281 phenotyped females and 304 phenotyped males that hatched on Mandarte were 0.36 ± 0.20 SD and 0.37 ± 0.19 SD, respectively. In (B), black, dark gray, mid gray, light gray, and white denote broods that contained 4, 3, 2, 1, and 0 extra‐pair offspring, respectively, within each brood size. One brood of five offspring (four extra‐pair) is not depicted.

The dataset comprised 1177 broods and associated observations of extra‐pair paternity spanning 1993–2016 (mean 49 ± 19 SD broods per year, range 16–80, Fig. 1, Supporting Information S3). These broods were produced by 295 females and reared by 309 socially paired males, forming 539 different social pairings (Supporting Information S3). The mean extra‐pair paternity rate was 0.288, but varied substantially among broods (Fig. 2).

A total of 31 immigrant song sparrows arrived on Mandarte during 1989–2016, comprising 17 females and 14 males. Of these, 14 female immigrants produced at least one brood during 1993–2016 and hence had an observed extra‐pair reproduction phenotype (58 broods observed in total), whereas 12 female immigrants contributed ≥1 female and/or male offspring with an observed phenotype. Overall, all 17 female immigrants contributed to observed phenotypes either directly and/or through offspring. In contrast, only five male immigrants reared at least one brood during 1993–2016 and hence had an observed paternity phenotype (28 broods in total), and one additional male immigrant contributed offspring with observed phenotypes. The other eight recent male immigrants did not breed, and therefore had no direct impact on any genetic characteristics of the focal population.

The defined immigrant genetic group comprising phantom parents of the 23 contributing recent immigrants made a substantial collective expected contribution to the phenotyped individuals’ genomes. Specifically, the mean immigrant genetic group contributions (*q*_i_) were 0.39 ± 0.24 SD and 0.38 ± 0.20 SD across all 295 phenotyped females and 309 phenotyped males, respectively (Fig. 2, Supporting Information S2). Recent immigrants therefore contributed over one‐third of each focal individual's genome on average, but with substantial among‐individual variation (Fig. 2). The pruned pedigree contained 738 individuals, with 32 defined founders. Mean pairwise coefficient of kinship between all phenotyped individuals was 0.069 ± 0.041 SD, providing substantial power for quantitative genetic analyses (Supporting Information S2).

The genetic groups animal model estimated moderate additive genetic variances, and hence heritabilities, in female extra‐pair reproduction (Var(*a*_f_)) and male paternity loss (Var(*a*_m_), Table 1), concurring with previous analyses (Reid et al. 2014a). However, interestingly, the posterior mean slopes of the regressions on immigrant genetic group contributions, and hence the estimated genetic group effects *g*_f_ and *g*_m_, were negative for both traits (Table 1). The 95% CI for female extra‐pair reproduction (*g*_f_) overlapped zero, but that for male paternity loss (*g*_m_) scarcely did, and ∼97% of posterior density was negative (Table 1). This implies that immigrant song sparrows to Mandarte (of both sexes) had lower mean breeding values for male paternity loss, and may also have had somewhat lower mean breeding values for female extra‐pair reproduction, than the defined founder population. Back‐transformations showed that the estimated effects are biologically substantial: a population with mean immigrant genetic group coefficient *q* = 0.37 would experience a predicted decrease in extra‐pair paternity rate from 0.37 (95% CI 0.10–0.63) to 0.18 (95% CI 0.06–0.28) compared to an otherwise identical population with no immigration and hence *q* = 0 (posterior mean difference: 0.19, 95% CI 0.02–0.37).

The posterior mean cross‐sex covariance in breeding value for female extra‐pair reproduction and male paternity loss (cov(*a*_f_,*a*_m_)) was negative, with a 95% CI that only marginally overlapped zero (∼95% of posterior density was negative, Table 1). This translates into a posterior mean cross‐sex genetic correlation of –0.51 (Table 1). This implies that female song sparrows with high breeding value for extra‐pair reproduction have male relatives with high breeding value for paternity success (i.e., liability to sire offspring in broods they rear).

However, as both immigrant genetic group effects *g*_f_ and *g*_m_ were negative, the cross‐sex covariance in total additive genetic value (cov(*u*_f_,*u*_m_)) was less negative than that in breeding value (cov(*a*_f_,*a*_m_), Table 2, following eq. 3). This constituted approximately a 25% decrease in magnitude of the posterior mean, and corresponding decrease in the proportion of posterior density that was negative (Table 2).

**Figure 3:**
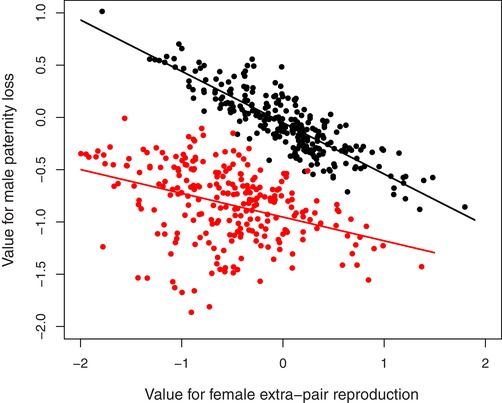
Illustration of posterior mean predicted breeding values (*a*_i_, black) and total additive genetic values (*u*_i_, red) for female liability for extra‐pair reproduction and male liability for paternity loss across 252 song sparrows hatched during 2003–2012. Lines represent regressions of male liability on female liability and are intended to illustrate the cross‐sex genetic correlation in breeding value (black) versus total additive genetic value (red). Note that breeding values are deviations from defined group means.

The variances in total additive genetic values for female extra‐pair reproduction (Var(*u*_f_)) and male paternity loss (Var(*u*_m_)) were slightly greater than the estimated variances in breeding values (Var(*a*_f_) and Var(*a*_m_), following eq. 2), representing approximately 10% and 40% increases, respectively (Tables 1 and 2). Consequently, the posterior mean cross‐sex correlation in total additive genetic value (cor(*u*_f_,*u*_m_)) was considerably less negative than that in breeding value (cor(*a*_f_,*a*_m_), Tables 1 and 2), representing approximately a 45% decrease. Figure 3 illustrates these effects across phenotyped individuals hatched during 2003–2012, showing that total genetic values are lower than breeding values, with reduced covariance.

Overall, the combination of the increases in Var(*u*_f_) and Var(*u*_m_) compared to Var(*a*_f_) and Var(*a*_m_), and the decrease in the (negative) magnitude of cor(*u*_f_,*u*_m_) compared to cor(*a*_f_,*a*_m_), meant that Var(*u*_T_) exceeded Var(*a*_T_) by approximately 50% (Tables 1 and 2, absolute posterior mean difference 0.53, mode 0.34, 95% CI 0.001–1.22). Recent immigration therefore substantially increased the total local additive genetic variance in liability for extra‐pair paternity that is available to generate an evolutionary response to local selection.

## Discussion

Gene flow resulting from successful immigration into any population might be expected to substantially alter means, variances, and cross‐sex covariances in breeding values and total additive genetic values for key traits that shape reproductive systems, and thereby alter forms of direct and indirect natural and sexual selection and evolutionary responses (e.g., Day 2000; Mead and Arnold 2004; Guillaume and Whitlock 2007; Long et al. 2012; Connallon et al. 2018). However, such quantitative genetic effects have not been explicitly quantified in wild animal populations experiencing natural immigration and resulting gene flow. We provide a tractable framework for estimating such effects from wild population data. Our analyses of extra‐pair paternity in socially monogamous song sparrows, a key reproductive trait that defies straightforward evolutionary explanation (Arnqvist and Kirkpatrick 2005; Reid et al. 2011, 2014a; Parker and Birkhead 2013), show that recent immigrants imported low breeding values for male paternity loss, and tended to import low breeding values for female extra‐pair reproduction, compared to the means estimated for the pre‐existing local population. Because recent immigrants contributed substantially to the genomes of contemporary individuals, their arrival will have substantially decreased genetic values for extra‐pair paternity, and hence increased the degree of reproductive fidelity among socially paired mates. Further, the immigrant effects weakened the negative cross‐sex covariance and correlation in total additive genetic values (*u*_i_) compared to those in breeding values (*a*_i_). They thereby increased the total genetic variance available to generate an evolutionary response to selection and potentially altered the effective magnitude of indirect selection on sex‐specific reproductive strategy. Immigration therefore substantially altered the phenotypic expression and evolutionary potential of a key reproductive outcome, extra‐pair paternity, that can affect population‐wide distributions of survival, reproductive success, relatedness, and parental care (Webster et al. 1995; Neff and Gross 2001; Sardell et al. 2011; Lebigre et al. 2012; Reid et al. 2016; Germain et al. 2018; Gow et al. 2019), and thereby shape overall social and reproductive systems.

The estimated differences in mean breeding values between recent immigrants and the defined founder population, which presumably includes genetic legacies of previous immigrants, could reflect multiple nonexclusive processes. At the meta‐population scale, recent (i.e., post‐1989) immigrants may originate from different source populations from previous immigrants with different mean breeding values for reproductive traits, and/or breeding values in immigrants’ source populations may have changed over time. There are still few rigorous data on extra‐pair paternity rates in other song sparrow populations. A North Carolina population showed lower mean phenotypic values than Mandarte (∼14% versus 28% chicks, ∼19% versus 44% broods; Krippel et al. 2017), but a Washington State population showed similar values to Mandarte (∼24% chicks, 36% broods; Hill et al. 2011). However, a meta‐analysis of *Emberizid* species inferred lower extra‐pair paternity rates at higher altitudes, especially at higher latitudes (Bonier et al. 2014). If such patterns have a genetic basis, the low breeding values of recent immigrants to Mandarte could potentially reflect increasing immigration from adjacent populations breeding at higher altitude in British Columbia's coastal mountains.

Alternatively or additionally, the subset of individuals that immigrate into Mandarte and successfully breed may have changed over time. Specifically, individuals with high breeding values for extra‐pair reproduction or paternity loss may now be less dispersive, or be more likely to fail to breed following dispersal and hence have zero genetic impact on recipient populations. Such associations between dispersal and reproductive traits have been extensively examined in the context of outcrossing versus selfing (Massol and Cheptou 2010; Hargreaves and Eckert 2014; Pannell 2015). However, there has been relatively little consideration of individual‐level (as opposed to population‐ or species‐level) associations between dispersal and key reproductive traits in self‐incompatible organisms, impeding assessment of how population connectivity affects mating system dynamics (Laloi et al. 2009; Ronce and Clobert 2012, but see Duckworth and Kruuk 2009). Dispersing individuals have been hypothesized to be more polyandrous in invertebrate systems with postmating dispersal; dispersers that colonize new areas could then produce half‐sib rather than full‐sib offspring and thereby reduce next‐generation inbreeding (Cornell and Tregenza 2007). However, there is scant empirical evidence for such dispersal‐polyandry covariances (Rafter et al. 2017; Rhainds 2017). There is, unsurprisingly, even less data on reproductive traits of dispersers that fail to breed. Eight (of 14) recent male immigrant song sparrows to Mandarte that did not pair or breed may be a nonrandom subset, and the frequency of such reproductive failure has increased in recent years as the local adult sex ratio has been more male‐biased on average (e.g., grand mean proportion males 1993–2005: 0.64; 1983–1992: 0.55, see also Smith and Arcese 1989). Although there is no currently tractable way to estimate breeding values for reproductive traits of new immigrants that never bred, the hypothesis that there is a negative additive genetic covariance between male liabilities for social pairing and for paternity loss conditional on pairing could in future be tested with the study population given the substantial observed variation in each trait.

Meanwhile, the observed differences in mean breeding values for extra‐pair paternity between recent immigrant song sparrows and defined founders may also reflect local evolutionary processes. Specifically, local direct or indirect selection, or drift operating in the small focal population, could have caused the genetic contributions of individuals with high breeding values for female extra‐pair reproduction and male paternity loss to increase across years. This hypothesis cannot be directly tested because breeding values for lineages that went locally extinct before 1993 cannot be evaluated. Future analyses with more years of data could test for increasing local mean breeding values since 1993. Indeed, current analyses suggested a slight increase in phenotypic extra‐pair paternity rate across years despite the negative effects of recent immigrants (Table 1), but did not aim to distinguish genetic change due to selection from environmental effects or drift. Given any local evolutionary increase, by importing low breeding values immigrants could help maintain substantial local total additive genetic variation in extra‐pair paternity and hence in the reproductive system.

However, it remains unclear how local selection could act to increase extra‐pair paternity; male paternity loss is likely to experience negative direct selection because low within‐pair paternity success is positively genetically correlated with low extra‐pair reproductive success (Reid et al. 2014c; Reid and Wolak 2018). Nevertheless, such local evolution could still occur if there were positive direct or indirect selection on female extra‐pair reproduction. Indeed, previous analyses suggested a weak positive genetic covariance between female extra‐pair reproduction and annual reproductive success (Reid 2012). Further, current analyses revealed a negative cross‐sex correlation in breeding values for female extra‐pair reproduction and male paternity loss (Table 1), which equates to a positive correlation between female extra‐pair reproduction and male paternity success. Such positive correlations could be created or magnified by assortative reproduction between polyandrous females and males with high siring success, and have been widely hypothesized to generate or exacerbate positive indirect selection on polyandry (Evans and Simmons 2008; Forstmeier et al. 2011, 2014; but see Bocedi and Reid 2015). Our current analyses provide clear evidence for such a positive correlation (although previous analyses showed that this does not translate into a positive genetic correlation between female extra‐pair reproduction and male lifetime reproductive success, Reid and Wolak 2018). Yet, our demonstration that the cross‐sex correlation in total additive genetic values was substantially weakened by the genetic effects of recent immigrants is equally important (Fig. 3). This result implies that immigration reduces the degree to which cross‐sex indirect selection could drive evolution of female extra‐pair reproduction.

Overall, our conceptual framework and empirical results have important general implications for quantifying and interpreting the magnitude of cross‐sex genetic correlations, and associated indirect selection and sexual conflict, in wild populations. In showing that gene flow resulting from natural immigration can increase total additive genetic variance, and alter the cross‐sex genetic correlation for key reproductive traits, they imply that micro‐evolutionary dynamics of perplexing reproductive traits and systems, including extra‐pair paternity and polyandry, might not be understandable solely in terms of local components of selection. There is increasing evidence of spatial variation and local adaptation in reproductive and sexually selected traits in diverse systems, including latitude (Taylor et al. 2014), and with different or changing local environmental conditions (Candolin 2019). In this context, our results demonstrate the need to place both theoretical and empirical studies of mating system evolution into appropriate spatially dynamic meta‐population contexts (e.g., Day 2000; Gosden and Svensson 2008; Ronce and Clobert 2012; Connallon et al. 2018), and we provide a tractable analytical approach with which to do so.

Associate Editor: A. Charmantier

## Supporting information


**Appendix S1**. Definitions and derivations.
**Appendix S2**. Details of quantitative genetic analyses.
**Appendix S3**. Additional summaries of dataset and results.
