# Supplementary material for: Recent immigrants alter the quantitative genetic architecture of paternity in song sparrows
Source: Evol Lett. 2020 Feb 25;4(2):124–36. doi: 10.1002/evl3.162 (PMC7156105; doi:10.1002/evl3.162)
Supplement: Supplementary file 1 — Appendix S1. Definitions and derivations. Appendix S2. Details of quantitative genetic analyses. Appendix S3. Additional summaries of dataset and results. [file EVL3-4-124-s001.docx]

**Supporting Information**

**Recent immigrants alter the quantitative genetic architecture of paternity in song sparrows**

Jane M. Reid and Peter Arcese

**Appendix S1. Definitions and derivations**

Full detailed explanations of ‘genetic groups animal models’, including ‘how to’ tutorials, are provided in Wolak and Reid (2017). Summary definitions of key terms are below. Key estimated quantities are also defined in main Tables 1 and 2.

***a*_i_**: individual *i*’s breeding value for a focal trait (i.e. the sum of the average additive allelic effects of an individual’s genotype, defined with respect to a specified reference population, and quantified as a deviation from the reference population’s mean genotypic value).

***q*_i_**: the proportion of individual *i*’s genome that is expected to have originated from the defined immigrant genetic group (i.e. individual genetic group coefficient). Can be straightforwardly calculated from complete pedigree data.

***g***: the genetic group effect, defined as the difference in mean additive genetic effect between the founder and immigrant genetic groups. Treated as a population-level fixed effect, most straightforwardly estimated as the slope of a regression of each focal trait on individual *q*_i_ fitted within a ‘genetic groups animal model’.

***u*_i_**: individual *i*’s total additive genetic value for a focal trait, encompassing its breeding value and proportional genetic group effect, where *u*_i_ = *a*_i_ + *g*·*q*_i_ (Equation 1).

**Var(a)**: Additive genetic variance (i.e. estimated variance in *a*_i_).

**Var(u)**: Variance in total additive genetic value (i.e. estimated variance in *u*_i_).

**Var(q)**: Estimated variance in *q*_i_.

**cov(*a*_f_,*a*_m_)** and **cor(*a*_f_,*a*_m_)**: Cross-sex additive genetic covariance and correlation (i.e. estimated covariance and correlation in *a*_i_).

**cov(*u*_f_,*u*_m_)** and **cor(*u*_f_,*u*_m_)**: Cross-sex covariance and correlation in total additive genetic value (i.e. estimated covariance and correlation in *u*_i_).

**Var(*a*_T_)**: total additive genetic variance in an ‘emergent’ trait, calculated as Var(*a*_T_) = Var(*a*_f_) + 2cov(*a*_f_,*a*_m_) + Var(*a*_m_) (Equation 4), where Var(*a*_f_) and Var(*a*_m_) are the additive genetic variances in the female and male trait respectively, and cov(*a*_f_,*a*_m_) is the cross-sex additive genetic covariance.

**Var(*u*_T_)**: total variance in total additive genetic value in an ‘emergent’ trait, calculated as Var(*u*_T_) = Var(*u*_f_) + 2cov(*u*_f_,*u*_m_) + Var(*u*_m_) (Equation 5), where Var(*u*_f_) and Var(*u*_m_) are the variances in additive genetic values in the female and male trait respectively, and cov(*u*_f_,*u*_m_) is the cross-sex covariance in total additive genetic value.

**Derivation of the variance in total additive genetic value Var(*u*) given an estimate of the variance in breeding value (additive genetic variance, Var(*a*)).**

General expressions for variances (σ^2^) of functions are:

σ^2^_bX_ = b^2^σ^2^_X_, where X is a variable and b is a constant.

σ^2^_X+Y_ = σ^2^_X_ + σ^2^_Y_, where X and Y are independent variables.

Hence, since *u*_i_ = *a*_i_ + *gq*_i_ (main Equation 1) where *g* is a constant (i.e. a fixed between group difference) and *q*_i_ and *a*_i_ are independent variables,

σ^2^_u_ = σ^2^_a_ + *g*^2^σ^2^_q_

Hence, an estimate of the variance in total additive genetic value Var(*u*) can be calculated from estimates of the additive genetic variance (Var(*a*)), genetic group effect (*g*) and the variance in individual genetic group coefficients (Var(*q*)) as:

Var(*u*) = Var(*a*) + *g*^2^·Var(*q*) Main Equation 2

This expression assumes that the covariance between *q*_i_ and *a*_i_ (i.e. σ(*q*_i_, *a*_i_)) is zero, which should hold true since values of *a*_i_ are estimated as deviations from the genetic group mean.

**Derivation of the cross-sex covariance in total additive genetic value cov(*u_f_,u_m_*) given an estimate of the cross-sex covariance in breeding value (additive genetic covariance, cov(*a_f_,a_m_*)).**

A general expression for the covariance (σ) between functions is:

σ_(X+Y,Z)_ = σ_(X,Z)_ + σ_(Y,Z)_, where X, Y and Z are independent variables.

Hence, since *u*_fi_ = *a*_fi_ + *g_f_q*_i_ and *u*_mi_ = *a*_mi_ + *g_m_q*_i_ where *g* is a constant (i.e. a fixed between group difference) and *q*_i_ and *a*_i_ are independent variables and subscripts f and m denote female and male traits respectively,

σ_uf,um_ = σ(*g_f_q*_i_, *g_m_q*_i_ + *a*_mi_) + σ(*a*_fi_, *g_m_q*_i_ + *a*_mi_)

σ_uf,um_ = σ(*g_f_q*_i_, *g_m_q*_i_) + σ(*g_f_q*_i_, *a*_mi_) + σ(*a*_fi_, *g_m_q*_i_) + σ(*a*_fi_, *a*_mi_)

In general, σ_(bX+cY)_ = b.c.σ_(X,Y)_

Hence σ_uf,um_ = *g_f_g_m_*σ(*q*_i_, *q*_i_) + *g_f_*σ(*q*_i_, *a*_mi_) + *g_m_*σ(*a*_fi_, *q*_i_) + σ(*a*_fi_, *a*_mi_)

Then, since σ(*q*_i_, *a*_i_) ≈ 0 (see above),

σ_uf,um_ = σ(*a*_fi_, *a*_mi_) + *g_f_g_m_*σ(*q*_i_, *q*_i_)

σ_uf,um_ = σ(*a*_fi_, *a*_mi_) + *g_f_g_m_*σ^2^(*q*_i_)

Hence, an estimate of the covariance in total additive genetic effects cov(*u*_f_,*u*_m_) can be calculated from estimates of the additive genetic covariance cov(*a*_f_,*a*_m_), genetic group effects (*g*_f_ and *g*_m_) and the variance in genetic group coefficients Var(*q*) as:

cov(*u*_f_,*u*_m_) = cov(*a*_f_,*a*_m_) + *g*_f_·*g*_m_·Var(*q*) Main Equation 3

These expressions clarify the ways in which immigration can cause the variance and covariance in total additive genetic values (*u_i_*) to differ from the variance and covariance in breeding values (*a_i_*).

Variance:

Since *g*^2^, Var(*q*) and Var(*a*) cannot be negative, the variance in total additive genetic values Var(*u*) will exceed the additive genetic variance Var(*a*) as long as there is some variance in immigrant genetic group contributions among population members (hence Var(*q*) ≠ 0) and mean breeding value differs (in either direction) between the native and immigrant populations (hence *g* ≠ 0).

Covariance:

If the additive genetic covariance cov(*a*_f_,*a*_m_) is positive, then the covariance in total additive genetic values cov(*u*_f_,*u*_m_) will be greater if the two genetic group coefficients *g*_f_ and *g*_m_ are both non-zero and have the same sign (i.e. both positive or both negative) and Var(*q*) ≠ 0. However cov(*u*_f_,*u*_m_) will be smaller than cov(*a*_f_,*a*_m_), and potentially negative, if *g*_f_ and *g*_m_ have different signs. Similarly, if cov(*a*_f_,*a*_m_) is negative, then cov(*u*_f_,*u*_m_) will be more negative if the two genetic group coefficients *g*_f_ and *g*_m_ have different signs, but less negative, and potentially positive, if they have the same sign.

Note that the derived expressions apply to estimates of V(*a*_f_), V(*a*_m_) and cov(*a*_f_,*a*_m_) obtained from standard genetic groups animal models, which assume that these quantities are the same for all genetic groups (Wolak and Reid 2017; Muff *et al.* 2019). Analogous expressions can be readily derived for the situation where V(*a*_f_), V(*a*_m_) and cov(*a*_f_,*a*_m_) differ among genetic groups (e.g. given *u*_i_ = *a_1_*_i_ + *a_2_*_i_ + *gq*_i_, where *a_1_*_i_ and *a_2_*_i_ are individual *i*’s partial breeding values with respect to each of two genetic groups, Muff *et al.* 2019). However, in practice, such parameters may rarely be estimable from wild population data with useful precision. Otherwise, the currently derived expressions are general, and apply to any two defined genetic groups, and are not specific to the case of founders and subsequent immigrants. However, in the current context, they allow calculation of Var(*u*_f_), Var(*u*_m_) and cov(*u*_f_,*u*_m_) for any hypothesised variance in admixture Var(*q*) from animal model estimates of Var(*a*_f_), Var(*a*_m_), cov(*a*_f_,*a*_m_), *g*_f_ and *g*_m_. Simulations confirmed that they yield unbiased estimates of these quantities. Such calculations facilitate unbiased estimation and statistical inference, but assume that Var(*a*_f_), Var(*a*_m_), cov(*a*_f_,*a*_m_), which are estimated for the defined founder and immigrant base populations, do not change over subsequent generations though which admixture occurs. This assumption is common to many evolutionary quantitative genetic projections, and may approximately hold over a relatively small number of generations, especially given weak selection on highly polygenic traits (e.g. Mead and Arnold 2004; Connallon and Hall 2016; Barton *et al.* 2017).

In principle, an alternative approach to estimate Var(*u*_f_), Var(*u*_m_) and cov(*u*_f_,*u*_m_) across any desired set of individuals, for example a contemporary population of admixed individuals, is to compute each individual’s value of *u*_i_ for each trait (Equation 1) and then compute the variances and covariance across individuals. This approach relaxes the assumption that key quantitative genetic parameters do not change, but may be more susceptible to bias due to the difference between true and predicted breeding values.

**Appendix S2. Details of quantitative genetic analyses**

**Animal model specifications**

In general, univariate animal models can be fitted to estimate sex-specific genetic and environmental components of variation in any ‘emergent’ trait that results from the joint actions of interacting females and males or other individuals, and hence estimate the cross-sex genetic covariance (and hence correlation). This approach has been applied to several traits that can be envisaged as the joint outcome of female and male effects (e.g. breeding date, Germain *et al.* 2016; inbreeding, Wolak and Reid 2016; divorce, Germain *et al.* 2018; and see Bijma *et al.* 2007 and Bijma 2011 for detailed explanation of this approach in the context of animal breeding).

The current univariate animal model considered the degree of extra-pair paternity as the focal jointly-expressed trait, and simultaneously estimated additive genetic variance in female liability for extra-pair reproduction and male liability for paternity loss, and the additive genetic covariance between the two. The model was of the form:

**Y = Xβ + Z_A.Fem_a_Fem_ + Z_A.Male_a_Male_ + Z_PI.Fem_PI_Fem_ + Z_PI.Male_PI_Male_ + Z_Pair_Pair + Z_Year_Year + e**

where **Y** is the probability that an offspring will be an extra-pair offspring versus a within-pair offspring on the logit scale. The vector of observed phenotypes was assumed to be a (overdispersed) binomial variable with the numbers of extra-pair and total offspring observed within each brood as binomial numerator and denominator (giving a single observed phenotype per brood) and probability equal to the inverse logit of **Y**. **X** and **Z** are appropriate design matrices respectively linking fixed and random effects to individuals. Overall, this model estimates female and male effects on the relative odds that a jointly reared offspring will be an extra-pair versus within-pair offspring.

**β** denotes vectors of fixed effects. These comprised year (since 1993), male age and, of particular importance for the current analyses, female and male immigrant genetic group coefficients *q*_i_. The resulting regressions on *q*_i_ generate a ‘genetic groups animal model’, and allow estimation of the fixed genetic group effect *g*. Note that fitting an animal model without genetic groups when there is in fact structure in the base population may yield biased estimates of means and variances in *a*_i_ (Wolak and Reid 2017).

**a_Fem_** and **a_Male_** are vectors of additive genetic values (i.e. breeding values) for female and male liabilities respectively, whose variances and cross-sex covariance are to be estimated. The covariance structure is specified as a Kronecker product of the general form **A ⊗ G**. This creates a block matrix containing all pairwise products of elements in **A** and **G**, where **A** is the matrix of additive genetic relationships between individuals (i.e. the numerator relationship matrix) and **G** is the additive genetic variance-covariance matrix for all females and males. Because females and males can share genetic effects, the cross-sex additive genetic covariance for any observed emergent trait is estimable from the probability that opposite-sex individuals share alleles identical by descent (as specified by the relationships described in **A**) within the univariate model. Because the Kronecker product equates to the total variance-covariance matrix of additive genetic random effects, the estimated genetic covariance equates to the cross-sex additive genetic covariance between female and male liabilities. Note that such covariances refer to associations across opposite sex relatives, not solely across socially-paired mates (Reid *et al.* 2014a).

**PI_Fem_** and **PI_Male_** are vectors of random permanent individual effects for the female that produced each brood and her socially paired male respectively. These effects could include permanent environmental effects and/or non-additive genetic effects. Since female liability for extra-pair reproduction and male liability for paternity loss are sex-limited traits, individuals cannot express both. The variances of **PI_Fem_** and **PI_Male_** are therefore independent, with zero permanent individual covariance across the sexes.

**Pair** and **Year** are vectors of random pair and year effects, thereby accounting for any positive correlations across multiple broods reared by individual pairs and within years, thereby ensuring independence of residuals across observed broods. Consequently, there is a single independent residual **e** for each observed brood in **Y**, meaning that there is no identifiable residual covariance between female and male effects within broods. This does not imply that additional brood-specific random effects on female and male liabilities are not correlated within individual broods, but any such covariance cannot be estimated separately and is subsumed without bias within the total estimated residual variance (thereby accounting for overdispersion).

**R code**

*##Prior*

*prior = list(R = list(V = 1, nu = 0.002), G = list(G1=list(V=diag(2),nu=2,alpha.mu=c(0,0),*

*alpha.V=diag(2)*1000), G2 = list(V = 1, nu = 1, alpha.mu = 0, alpha.V = 1000), G3 = list(V = 1, nu = 1, alpha.mu = 0, alpha.V = 1000), G4 = list(V = 1, nu = 1, alpha.mu = 0, alpha.V = 1000), G5 = list(V = 1, nu = 1, alpha.mu = 0, alpha.V = 1000)))*

*##Main model*

*model <- MCMCglmm(cbind(number.epo, number.wpo) ~ 1 + q.female + q.male + male.age + year.cont,*

*random = ~ str(female.animal + male.animal) + female.ID + male.ID + pair.ID + year.ID,*

*family = "multinomial2", data = data, prior = prior,*

*ginverse=list(female.animal=inv.A, male.animal=inv.A),*

*pr = TRUE,*

*burnin = 5000, nitt = 2005000, thin=1000)*

*##Results extraction*

*#Main fixed effects*

*plot(model$Sol[,1:5])*

*posterior.mode(model$Sol[,1:5])*

*HPDinterval(model$Sol[,1:5], 0.95)*

*autocorr(model$Sol[,1:5])*

*#(Co)variance components*

*plot(model$VCV)*

*posterior.mode(model $VCV)*

*HPDinterval(model $VCV, 0.95)*

*autocorr(model $VCV)*

*summary(model)*

Here, *number.epo* and *number.wpo* are the numbers of extra-pair and within-pair offspring observed in each brood included in the analysis. *cbind(number.epo, number.wpo)* is standard code to define a single binomial dependent variable.

*q.female* and *q.male* are the immigrant genetic group coefficients (*q*_i_) of the socially-paired female and male that reared each brood.

*male.age* is the age of each focal male (in years).

*year.cont* and *year.ID* are the observation year as a continuous variable (since 1993) and a factor respectively.

*female.animal* and *male.animal* identify the female and male that reared each brood and link them to the inverse A matrix. The ‘str’ function generates the direct cross-product, allowing estimation of the cross-sex genetic covariance within the univariate model.

*inv.A* is the inverse A matrix generated by *inv.A <- inverseA(ped)$Ainv*, where *ped* is the pedigree pruned to all phenotyped individuals and all their known ancestors.

*pr = TRUE* saves predicted breeding values *a*_i_ for each individual in the pedigree. These are required to directly calculate each individual’s total additive genetic value (*u*_i_), if this is desired.

*female.ID, male.ID* and *pair.ID* are identities of each focal female, male and social pairing.

*burnin, nitt and thin* are model specifications: models were run for 2005000 iterations with burn-in 5000 and thinning interval 1000, yielding 2000 samples of the posterior distribution. Autocorrelation among samples was small (<0.05 for all parameters). Visual inspection, and assessment of results across three independent chains, confirmed convergence.

Models were run with default priors on fixed effects (with mean zero and large variance), and parameter expanded priors on all (co)variance components. The primary formulation generates a weakly informative prior on the cross-sex genetic correlation (cor(*a*_f_,*a*_m_)), with greater density near -1 and 1. This implies that the posterior distribution, with posterior mean ~-0.5, must be substantially informed by the data. Indeed, posterior distributions remained similar when models were rerun with different prior formulations (as in Wolak *et al.* 2018).

The first five reported fixed effects solutions are the intercept, and the slopes of the regressions on female and male genetic group contributions (*g*_f_ and *g*_m_), male age and year respectively. The values of *g*_f_ and *g*_m_ are of primary interest, and estimate the difference in mean breeding value between recent immigrants and defined founders for female extra-pair reproduction and male paternity loss respectively. The remaining reported solutions give the breeding values (*a*_i_) for female extra-pair reproduction and male paternity loss estimated for all individuals (of both sexes) included in the pruned pedigree. Note that genetic groups animal models can alternatively be specified to directly yield estimates of *u*_i_ rather than *a*_i_ (Wolak and Reid 2017).

Previous detailed analyses yielded no evidence of substantial effects of the identities of an individual’s mother or social father on female extra-pair reproduction or male paternity loss (Reid *et al.* 2014a), and hence no evidence that estimated additive genetic variances could be inflated by unmodelled parental effects that could increase phenotypic resemblance among (half)-siblings. Consequently, such parental effects were not further considered in current analyses.

Current analyses assume that additive genetic variances and covariance in female extra-pair reproduction and male paternity loss are the same in the defined immigrant and founder genetic groups (Wolak and Reid 2017). While it is possible to relax this assumption, at least assuming no segregation variance (Muff *et al.* 2019), such extensions double the number of genetic (co)variances to be estimated (given two genetic groups). Exploratory analyses showed that the song sparrow data were insufficient to estimate group-specific additive genetic variances and covariances with useful precision. The values estimated across both genetic groups are likely to represent means, perhaps slightly weighted towards the defined founder group which is slightly larger (phantom parents of 32 versus 23 individuals, e.g. Muff *et al.* 2019).

Current analyses also assume that mean breeding values for female extra-pair reproduction and male paternity loss do not differ between female and male immigrants. In principle, separate genetic groups could be defined for female and male immigrants, thereby allowing estimation of the difference in mean breeding value for any focal trait between immigrants of difference sexes. However, such effects could not be estimated with useful precision with the song sparrow data. Exploratory analyses, so far as data allowed, suggested broadly similar effects of immigrants of both sexes.

In general, animal model estimates of additive genetic variances can be biased by unmodelled inbreeding depression (Reid and Keller 2010). However, previous analyses showed no evidence of inbreeding depression in female extra-pair reproduction or male paternity success per brood in the focal population (Reid *et al.* 2011, 2014a). Current models therefore did not model inbreeding depression (which can be achieved by fitting regressions on female and male coefficients of inbreeding). This allowed inclusion of phenotypic observations of immigrants, whose coefficients of inbreeding are undefined relative to the local pedigree base population. Main results remained quantitatively similar when analyses were rerun including fixed effects of an individual’s immigrant status (i.e. immigrant or Mandarte-hatched), and such immigrant effects were estimated to be small.

The observed degree of extra-pair paternity for any focal social pair may additionally depend on genetic and environmental effects of the potential extra-pair males (and females) with which a focal pair interact. However, such interactions might typically involve several individuals, meaning that genetic effects of any one extra-pair individual on paternity outcomes for a focal pair might be relatively small.

**Pedigree and pedigree analyses**

The genetic parentage data were used to compile a complete genetic pedigree for 1993-2016, with all individuals assigned to their true genetic parents with very high individual-level confidence (Reid *et al.* 2014b; Nietlisbach *et al.* 2017). Pedigree data derived from observed social parentage (i.e. parents that reared each brood) are available for 1975-1992. Although these data presumably contain paternity error stemming from unknown extra-pair reproduction, all maternal links and ca.72% of paternal links will be correct assuming similar extra-pair paternity rates to those observed from 1993. They therefore contain useful information regarding relatedness among individuals that bred from 1993 onwards. We therefore grafted the genetic pedigree for 1993-2012 onto the social pedigree for 1975-1992, thereby relaxing the alternative assumption that 1993 breeders were unrelated (Reid *et al.* 2014b). To further minimise pedigree error, individuals hatched during 1991-1992 that bred subsequently were genotyped to verify the paternity of individuals that contributed phenotypic data (Reid *et al.* 2014b).

The full pedigree was pruned to individuals that were informative for current analyses, namely 604 phenotyped adults (295 females and 309 males) and their known ancestors using the *prunePed* function in R packages MCMCglmm (Hadfield 2010) or nadiv (Wolak 2012). The pruned pedigree comprised 738 individuals, including 32 individuals that had unknown parents but were not recent immigrants, and hence comprised the defined founder population. In practice, key results remained similar when the founder population was re-defined as individuals present in the population in 1989 (i.e. immediately preceding the period through which genetic paternity was ascertained and hence the phenotypes of female extra-pair reproduction and male paternity loss observed). Figure S1 shows the distribution of pairwise coefficient of kinship between all 604 phenotyped individuals. The moderately high variance generates power for quantitative genetic analyses. The phantom parents of all recent immigrants and defined founders were assumed to be unrelated and outbred (as for all pedigree-based animal model analyses). Since immigrants were relatively infrequent and few immigrant males bred there were no immigrant-immigrant pairings.

**Figure S1.** Distribution of pairwise coefficient of kinship (*k*) among all 604 phenotyped individuals (excluding an individual’s *k* with itself). This yields N = n^2^-n values, where n=604, giving N=364212. Overall, mean *k* was 0.069±0.041SD, variance 0.002, interquartile range 0.046-0.084, range 0.000-0.424. For reference, values of *k*=0.25 and *k*=0.125 indicate outbred first-order and second-order relatives respectively. Values of zero typically represent pairwise comparisons involving immigrants.


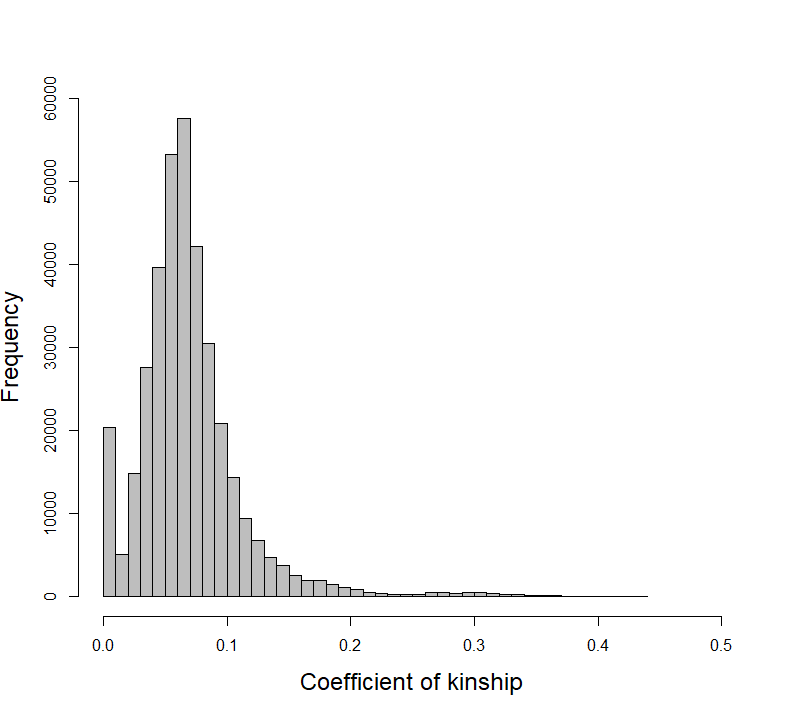


Mean *k* across the 539 social pairings with observed phenotypes was 0.083±0.056SD (minimum 0.000, maximum 0.357). Such variation in relatedness among pairs facilitates estimation of direct and associative genetic effects.

**Genetic group contributions**

The proportion of each individual’s genome (*q*_i_) that is expected to have originated from the defined immigrant genetic group was calculated from the pruned pedigree using the *ggcontrib* function in R package nadiv (Wolak 2012; full details of methods are in Wolak and Reid 2017).

Values of *q*_i_ increased across individuals whose phenotypes for female extra-pair reproduction and male paternity loss were observed during 1993-2016 (Figure S2). This reflects increasing introgression of genes originating in recent immigrants. Female and male genetic group contributions (*q*_i_) were weakly positively correlated across all observed broods (Pearson correlation coefficient *r*_p_ = 0.29). Further, female and male *q*_i_ were both positively corrected with year during 1993-2016 (*r*_p_=0.53 for both sexes, across all observed broods). However, key results remained similar when animal models were refitted excluding the regression on year (i.e. *year.cont*).

**Figure S2.** Distribution of the proportion of each individual’s genome (*q*_i_) that is expected to have originated from the defined immigrant genetic group across adult song sparrows that reared observed broods during 1993-2016. Boxplots show the median (black line), first and third quantiles (box limits), 1.5 times the interquartile range (whiskers) and outliers (points) across both sexes combined. Outlying points with values of *q*_i_ = 1 represent recent immigrants.


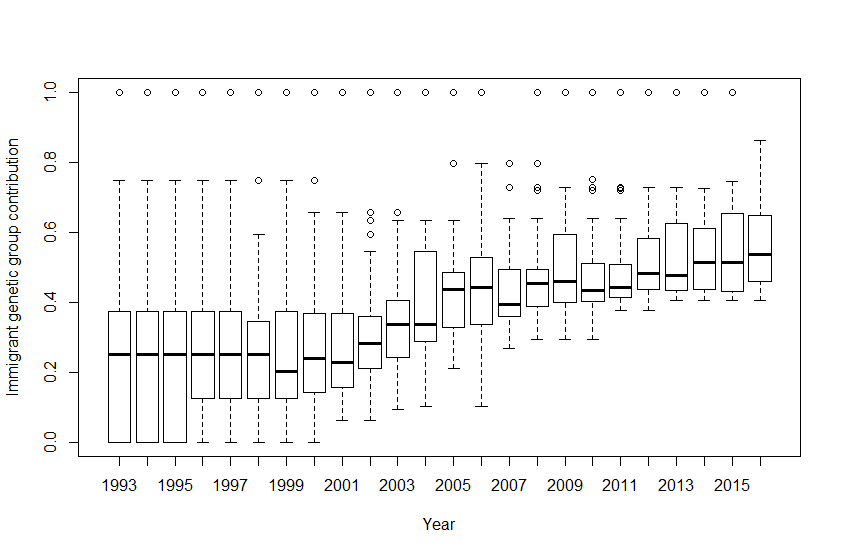


**Table S1.** Mean and variance of genetic group contribution (*q*_i_) across adult song sparrows that produced all broods for which a phenotype (i.e. female extra-pair reproduction and male paternity loss) was observed in each year during 1993-2016.

| **Year** | **1993** | **1994** | **1995** | **1996** | **1997** | **1998** | **1999** | **2000** |
| --- | --- | --- | --- | --- | --- | --- | --- | --- |
| **Mean** | 0.22 | 0.23 | 0.24 | 0.26 | 0.29 | 0.29 | 0.30 | 0.33 |
| **Variance** | 0.06 | 0.05 | 0.04 | 0.05 | 0.05 | 0.06 | 0.05 | 0.08 |
| **Year** | **2001** | **2002** | **2003** | **2004** | **2005** | **2006** | **2007** | **2008** |
| **Mean** | 0.31 | 0.33 | 0.36 | 0.40 | 0.46 | 0.44 | 0.45 | 0.47 |
| **Variance** | 0.06 | 0.04 | 0.04 | 0.02 | 0.04 | 0.03 | 0.02 | 0.02 |
| **Year** | **2009** | **2010** | **2011** | **2012** | **2013** | **2014** | **2015** | **2016** |
| **Mean** | 0.53 | 0.48 | 0.51 | 0.52 | 0.55 | 0.55 | 0.58 | 0.56 |
| **Variance** | 0.03 | 0.02 | 0.03 | 0.02 | 0.03 | 0.02 | 0.03 | 0.02 |

**Appendix S3. Additional summaries of dataset and results**

**Table S2.** Number of broods for which an extra-pair paternity phenotype (i.e. female extra-pair reproduction and male paternity loss) was observed in each year during 1993-2016.

| **Year** | **1993** | **1994** | **1995** | **1996** | **1997** | **1998** | **1999** | **2000** |
| --- | --- | --- | --- | --- | --- | --- | --- | --- |
| **Broods** | 70 | 66 | 76 | 76 | 80 | 49 | 16 | 24 |
| **Year** | **2001** | **2002** | **2003** | **2004** | **2005** | **2006** | **2007** | **2008** |
| **Broods** | 20 | 43 | 48 | 31 | 57 | 54 | 47 | 54 |
| **Year** | **2009** | **2010** | **2011** | **2012** | **2013** | **2014** | **2015** | **2016** |
| **Broods** | 41 | 72 | 37 | 59 | 52 | 49 | 33 | 23 |

**Distributions of observed broods across individuals and pairings:**

Number of broods per individual female: 4.0±3.1SD (median 3, range 1-17).

Number of broods per individual male: 3.8±3.0SD (median 3, range 1-19).

Number of broods per pairing: 2.2±1.5SD (median 2, range 1-10).

**Number of observed pairings per individual:**

Females: 1.8±1.2SD (median 1, range 1-6).

Males: 1.7±1.1SD (median 1, range 1-7).

**Distribution of observed male age:**

Mean 2.7±1.5SD (median 2, range 1-9).

**Table S3.** Posterior mean, mode and 95% credible interval (95%CI) for permanent individual female, male, pair and year variances estimated from the genetic groups animal model. Other model estimates are in Table 1.

|  | Mean [mode] | 95%CI |
| --- | --- | --- |
| Female permanent individual variance | 0.41 [0.01] | <0.001 – 1.09 |
| Male permanent individual variance | 0.11 [0.002] | <0.001 – 0.42 |
| Pair permanent variance | 0.23 [0.01] | <0.001 – 0.77 |
| Year variance | 0.03 [<0.001] | <0.001 – 0.13 |

Note that since some individual females and male only formed one pairing, and only reared one observed brood, permanent individual, pair and residual variances are likely to be somewhat confounded. However, such effects are unlikely to affect main conclusions of current analyses regarding additive genetic variances and covariances (thoroughly explored by Reid *et al.* 2014a, and further confirmed through further analyses of the current dataset).

**References**

Barton, N.H., Etheridge, A.M. & Véber, A. (2017). The infinitesimal model: Definition, derivation, and implications. *Theor. Pop. Biol.* 118:50-73.

Bijma, P., Muir, W.M. & Van Arendonk, J.A.M. (2007). Multilevel Selection 1: Quantitative genetics of inheritance and response to selection. *Genetics* 175:277-288.

Bijma, P. (2011). A general definition of the heritable variation that determines the potential of a population to respond to selection. *Genetics* 189:1347-1359.

Germain, R.R., Wolak, M.E., Arcese, P., Losdat, S. & Reid, J.M. (2016). Direct and indirect genetic and fine-scale location effects on breeding date in song sparrows. *J. Anim. Ecol.* 85:1613-1624.

Germain, R.R., Wolak, M.E. & Reid, J.M. (2018). Individual repeatability and heritability of divorce in a wild population. *Biol. Let.* 14:20180061.

Hadfield, J.D. (2010). MCMC methods for multi-response generalized linear mixed models: the MCMCglmm R package. *J. Stat. Soft*. 33:1-22.

Muff, S., Niskanen, A.K., Saatoglu, D., Keller, L.F. & Jensen, H. (2019). Animal models with group-specific additive genetic variances: extending genetic group models. *Genet. Sel. Evol.* 51:7.

Nietlisbach, P., Keller, L.F., Camenisch, G., Guillaume, F., Arcese, P., Reid, J.M. & Postma, E. (2017). Pedigree-based inbreeding coefficient explains more variation in fitness than heterozygosity at 160 microsatellites in a wild bird population. *Proc. R. Soc. B* 284:20162763.

Reid, J.M., Arcese, P., Sardell, R.J. & Keller, L.F. (2011). Heritability of female extra-pair paternity rate in song sparrows (*Melospiza melodia*). *Proc. R. Soc. B* 278:1114-1120.

Reid, J.M., Arcese, P., Keller, L.F. & Losdat, S. (2014a). Female and male genetic effects on offspring paternity: additive genetic (co)variances in female extra-pair reproduction and male paternity success in song sparrows (*Melospiza melodia*). *Evolution* 68:2357–2370.

Reid, J.M., Keller, L.F., Marr, A.B., Nietlisbach, P., Sardell, R.J. & Arcese, P. (2014b). Pedigree error due to extra-pair reproduction substantially biases estimates of inbreeding depression. *Evolution* 68:802–815.

Reid, J.M. & Keller, L.F. (2010). Correlated inbreeding among relatives: occurrence, magnitude and implications. *Evolution* 64:973-985.

Wolak, M.E. (2012). nadiv: an R package to create relatedness matrices for estimating non-additive genetic variances in animal models. *Methods Ecol. Evol.* 3:792–796.

Wolak, M.E. & Reid, J.M. (2016). Is pairing with a relative heritable? Estimating female and male genetic contributions to the degree of biparental inbreeding in song sparrows (*Melospiza melodia*). *Am. Nat.* 187:736-752.

Wolak, M.E. & Reid, J.M. (2017). Accounting for genetic differences among unknown parents in microevolutionary studies: How to include genetic groups in quantitative genetic animal models. *J. Anim. Ecol.* 86:7-20.
